# Chitosan–Aloe Vera Composition Loaded with Zinc Oxide Nanoparticles for Wound Healing: *In Vitro* and *In Vivo* Evaluations

**DOI:** 10.1049/2024/6024411

**Published:** 2024-05-15

**Authors:** Hasanain Adel Alawadi, Kamyab Andarzbakhsh, Ali Rastegari, Zohreh Mohammadi, Mehdi Aghsami, Fatemeh Saadatpour

**Affiliations:** ^1^Department of Pharmaceutics and Pharmaceutical Nanotechnology, School of Pharmacy, Iran University of Medical Sciences, Tehran, Iran; ^2^Department of Pharmaceutics, Faculty of Pharmacy, Tehran Medical Sciences, Islamic Azad University, Tehran, Iran; ^3^Department of Pharmacology and Toxicology, School of Pharmacy, Iran University of Medical Sciences, Tehran, Iran; ^4^Molecular Virology Lab, Department of Microbiology, School of Biology, College of Science, University of Tehran, Tehran, Iran

## Abstract

Global concerns due to the negative impacts of untreatable wounds, as well as the growing population of these patients, emphasize the critical need for advancements in the wound healing materials and techniques. Nanotechnology offers encouraging avenues for improving wound healing process. In this context, nanoparticles (NPs) and certain natural materials, including chitosan (CS) and aloe vera (AV), have demonstrated the potential to promote healing effects. The objective of this investigation is to assess the effect of novel fabricated nanocomposite gel containing CS, AV, and zinc oxide NPs (ZnO NPs) on the wound healing process. The ZnO NPs were synthesized and characterized by X-ray diffraction and electron microscopy. Then, CS/AV gel with different ratios was prepared and loaded with ZnO NPs. The obtained formulations were characterized *in vitro* based on an antimicrobial study, and the best formulations were used for the animal study to assess their wound healing effects in 21 days. The ZnO NPs were produced with an average 33 nm particle size and exhibited rod shape morphology. Prepared gels were homogenous with good spreadability, and CS/AV/ZnO NPs formulations showed higher antimicrobial effects against *Staphylococcus aureus*, *Escherichia coli, and Pseudomonas aeruginosa*. The wound healing findings showed significant wound area reduction in the CS/AV/ZnO NPs group compared to negative control at day 21. Histopathological assessment revealed the advantageous impact of this formulation across various stages of the wound healing process, including collagen deposition (CS/AV/ZnO NPs (2 : 1), 76.6 ± 3.3 compared to negative control, 46.2 ± 3.7) and epitheliogenesis (CS/AV/ZnO NPs (2 : 1), 3 ± 0.9 compared to negative control, 0.8 ± 0.8). CS/AV gel-loaded ZnO NPs showed significant effectiveness in wound healing and would be suggested as a promising formulation in the wound healing process. Further assessments are warranted to ensure the robustness of our findings.

## 1. Introduction

Wounds are precisely classified as instances where the typical integrity of skin is disturbed, along with noticeable changes in the inherent function of skin layers [1]. The increasing number of patients with untreatable wounds, combined with its negative impact on economic and healthcare systems, presents a worldwide concern [2]. In a meta-analysis the pooled prevalence of chronic ulcers and leg ulcers has been estimated to be 2.21 per 1,000 and 1.51 per 1,000 population, respectively [3]. Healing of wounds is considered a multifaceted procedure with four distinct phases, including coagulation (also has been mentioned as hemostasis), inflammation, proliferation, and remodeling [4]. Factors that interfere with this process could disturb normal healing procedures as well as worsening the damage condition [5]. In this regard, certain natural materials, including chitosan (CS) and aloe vera (AV), have demonstrated the potential to promote healing effects by influencing the stages mentioned above [6–8].

CS is a cationic polysaccharide that consists of glucosamine and *N*-acetyl glucosamine units [9]. CS and its derivatives have shown high-potential wound healing efficiency. They exhibited interesting characteristics, including biocompatibility, antimicrobial effects, and hemostatic properties [10–12]. Studies have reported that CS could serve as a potential material for wound healing, which impacts all healing stages [13–15]. Due to the mentioned notable properties, CS is being employed in various drug delivery systems for wound healing [13]. In addition, AV, as an herbal extract, has been used by humans since ancient times. The bioactive materials that constitute AV are reported to have applications in different fields, encompassing the food industry, health care, and cosmetics [3]. There are evidences pointing to the ability of AV in improving the properties of other materials, including biocompatibility, stability, and other characteristics, which are appropriate for wound healing [16–18]. Studies have shown that AV can be employed as a material for wound healing in diabetic rats which promotes essential healing processes such as inducing proliferation of fibroblasts and also accelerating collagen synthesis [19, 20].

Nowadays, nanoparticles (NPs) have opened new sights in biomedical sciences and have been shown to promote wound healing properties [21–24]. Zinc oxide nanoparticles (ZnO NPs) are one of the materials that exhibited desirable characteristics, including low cytotoxicity, antimicrobial, and antioxidant activities [25–28]. Recently, reports have demonstrated that ZnO NPs can accelerate wound closure rates and regulate all wound healing stages [29–31]. In a recent study, a hydrogel containing CS, polyvinyl alcohol (PVA), and AV-incorporated ZnO NPs displayed suitable properties as a wound dressing nanocomposite. However, this study did not evaluate the efficacy of prepared hydrogel in animals and proposed that there is potential for such an evaluation to be conducted [17].

Although CS and AV have been studied as wound healing materials in various studies so far to our knowledge, no study has been investigating the potential of CS/AV gel contained ZnO NPs for wound healing in animal models. For this purpose, in this study, CS/AV gel loaded with ZnO NPs has been fabricated as a nanocomposite gel, characterized with X-ray diffraction analysis (XRD) as well as field-emission scanning electron microscopy (FESEM) and subsequently evaluated its *in vitro* and *in vivo* effectiveness in promoting wound healing.

## 2. Materials and Methods

### 2.1. Materials

Chitosan (high molecular weight) was obtained from Iran chitosan company (Iran). *Aloe vera* powder was purchased from Titrachem Company (Iran). Zinc acetate and Triton X-100 were provided from Samchun Pure Chemical Company (South Korea) and Sigma–Aldrich Company (USA), respectively. All additional reagents were acquired from Merck (Darmstadt, Germany).

### 2.2. Microorganism Strains

The LB broth and agar were the media used throughout the study for *Staphylococcus aureus* (ATCC 6538), *Escherichia coli* (ATCC 11079), and *Pseudomonas aeruginosa* (ATCC 27853) cultivation at 37°C.

### 2.3. Preparation of ZnO Nanoparticles

Fabrication of ZnO NPs was performed according to the previous study [32]. Briefly, a flask was filled with 25 mL of 0.2 N zinc acetate solution and positioned on a magnetic stirrer. In the following, 4.5 mL Triton X-100 was introduced, and the prepared mixture was incubated at room temperature overnight. The pH was adjusted to 9.0 using dropwise 0.1 M NaOH, and then the solution was stirred for 24 hr to facilitate the sedimentation. The solution was then subjected to centrifugation at an 8,000 rpm speed rate for 30 min. The sediment was subjected for a wash procedure with deionized water and then with absolute ethanol and dried in an oven at 60°C. Afterward, the dried precipitate was subjected to 1 hr of calcination in a furnace at 400°C, which led to a color change from white to gray.

### 2.4. Characterization of ZnO Nanoparticles

The crystallinity of ZnO NPs was investigated by employing XRD (D4-BRUKER), which was equipped with a Cu-K*a* source. Furthermore, FESEM (Hitachi S-4160, Germany) was used for the assessment of morphology and size of prepared NPs.

### 2.5. Preparation of Chitosan/Aloe Vera Gel Loaded with ZnO Nanoparticles

CS/AV gel has been fabricated with two different ratios (w/w) of CS/AV components (1 : 1 and 2 : 1). First, CS solution 2% (w/v) was prepared in aqueous acetic acid 1% (v/v). Then, AV powder was added to the CS solution and subjected to overnight stirring to prepare various ratios of CS/AV gel. For preparation of CS/AV gel loaded with ZnO NPs (CS/AV/ZnO), after preparation of the mentioned gel, 5 mg ZnO NPs (1 mg/mL) were added to the obtained gel and stirred at room temperature overnight to obtaining a homogenous gel.

### 2.6. Characterization of Formulated Gels

The prepared CS/AV gel contained ZnO NPs was characterized for homogeneity, appearance, spreadability, and pH value by using a digital pH meter. The spreadability of the prepared gel was investigated by placing the gel and the standard gel (commercial diclofenac gel) on the glass at an angle of 90°, and the descent of the gel from the top of the glass to the bottom was evaluated.

### 2.7. Antimicrobial Study

Growth inhibition assay was performed by the broth tube method in accordance with the Clinical and Laboratory Standards Institute guidelines (CLSI). Different formulations including AV gel, CS gel, ZnO NPs (1 mg/mL), CS/AV (1 : 1), CS/AV (2 : 1), CS/AV/ZnO (1 : 1), and CS/AV/ZnO (2 : 1) gels were prepared according to the Section 2.5. Then 100 *μ*L of standardized inoculate of each microorganism, including *S. aureus*, *E. coli*, and *P. aeruginosa* (106 cells/mL), was inoculated in each well at the final volume of 3 mL, then incubated at 37°C. For bacterial load reduction assessment, 10 *µ*L of the cultures were streaked on agar plates at a time interval of 0, 1, 2, 4, 8, 12, and 24 hr, and colony formation was calculated after 48 hr of incubation.

### 2.8. *In Vivo* Wound Healing Study

Three weeks male Wistar rats (weighting between 70 and 100 g) were acquired from the Pasteur Institute of Iran. The execution of the animal experiment was performed in adherence with the protocols of the Animal Ethics Committee of the Iran University of Medical Sciences (IR.IUMS.REC.1400.822). In order to investigate the wound healing efficiency of prepared gels, randomly 49 rats were divided into seven groups, including (1) a negative control group, which acute wounds have been created but no treatment will be given to them, (2) positive control group which receiving topical gel of phenytoin 1%, (3) treated group with 1 mg/mL ZnO NPs, (4) treated group with CS/AV gel (1 : 1), (5) treated group with CS/AV gel ratio (2 : 1), (6) treated group with CS/AV/ZnO gel ratio (1 : 1), (7) treated group with CS/AV/ZnO gel ratio (2 : 1). First, the rats were subjected to anesthesia by employing ketamine10% and sedaxyl 20 mg/mL (70 : 30). Subsequently, approximate 1 cm^2^ wounds were generated by removing a layer of skin from the shaved area. Wounded subjects were kept in standard laboratory conditions. The day of operation is considered as day zero. For investigation of wound healing efficiency, each group received 1 mL formulation every day for 21 days and then bandaged with sterile gauze and wound adhesive. The area of the wound was accurately calculated by using Image J software on 1, 7, 14, and 21 days.

### 2.9. Histological Analysis

After 21 days, five subjects from each group underwent euthanasia by employing spinal cord injury under anesthesia. Tissues were gathered and fixed in 10% formalin for 48 hr. Afterward, the tissues were subjected to paraffin and then sliced into 5 *µ*m sections. Sections were subjected to hematoxylin and eosin (H&E) staining, and an independent observer conducted the assessment by employing light microscopy (Olympus BX51; Olympus, Tokyo, Japan). The following factors have been investigated in all groups: epithelialization, infiltration of inflammatory cells, fibroplasia, and the formation of granulation tissue. Furthermore, the epithelialization has been studied using a 5-point scale, which is mentioned in the following: 0 (without new epithelialization), 1 (25%), 2 (50%), 3 (75%), and 4 (100%) of epithelialization. In addition, Masson's trichrome (MT) staining was used for collagen density calculation using computer software Image-Pro Plus® V.6 (Media Cybernetics, Inc., Silver Spring, USA).

### 2.10. Statistical Analysis

All data are presented as mean ± standard deviation. The results were analyzed by employing *t*-test and ANOVA (the significance level was set at 0.05) by GraphPad Prism software (version 5.04 for Windows, GraphPad Software, San Diego California USA, https://www.graphpad.com).

## 3. Results and Discussion

### 3.1. Characterization of ZnO Nanoparticles

**Figure 1:**
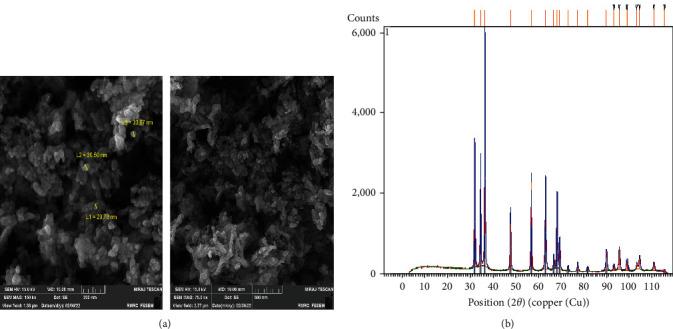
ZnO NPs characterization. FESEM image a different magnification (a) and XRD pattern (b) of ZnO NPs.

Characterization of ZnO NPs performed using XRD and FESEM methods. The obtained results are shown in Figure 1. The prepared ZnO NPs, with an average 33 nm particle size, exhibited rod-shaped morphology and homogenous structure. Besides, XRD analysis displayed the hexagonal structure of ZnO NPs crystallinity with peaks, which were indexed according to Joint Committee on Powder Diffraction Standards (JCPDS) card no. 96-230-0113. By employing the Scherrer equation, ZnO NPs displayed a 31.8 nm crystallite size [33].

### 3.2. Characterization of Gels

According to the obtained results (Table 1), all formulations appeared yellow and showed good homogeneity. Alvandi et al. [17] Indicated that introducing 5% AV to the combination of CS and PVA improved the mechanical properties compared to CS/PVA alone. They stated that this improvement might be due to the higher porosity obtained after adding AV [17]. In the current study, all formulations were without gritty particles, and no phase separation was observed. The spreadability of prepared gels was similar to standard gel and formulations showed good spreadability. Furthermore, the pH value of formulations was 5.5, which was acceptable for dermal applications.

### 3.3. Antimicrobial Study

**Figure 2:**
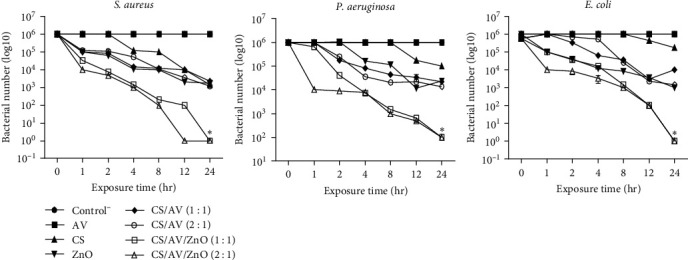
Growth inhibition assay of different formulations at different time intervals (0, 1, 2, 4, 8, 12, and 24 hr). Data are represented as mean ± SD (*n* = 3).  ^*∗*^ Denotes significant differences with *p*  < 0.0001.

The antimicrobial study was performed on different formulations against *S. aureus*, *E. coli*, and *P. aeruginosa*. According to the obtained results in Figure 2, the microbial count in CS/AV/ZnO gels with different ratios 1 : 1 and 2 : 1 was significantly decreased in comparison with the control group at each time point. The prepared gels, composed of chitosan, aloe vera, and ZnO nanoparticles, exhibited significant antibacterial activity against both Gram-positive and Gram-negative bacteria. The results of the current study are consistent with previous studies that reported an increased antimicrobial activity of chitosan–aloe vera (CS/AV) mixture compared to the individual components. The enhanced antimicrobial activity of CS/AV mixture could be related to the binding of chitosan molecules to the surface of the aloe vera molecules and making them more positively charged to enhance the antimicrobial activity of the mixture [34]. Furthermore, the obtained results demonstrated that the incorporation of ZnO NPs into the CS/AV gel could significantly improve the antibacterial activity of prepared gels which is in agreement with previous studies [17, 35]. In the prepared gels, ZnO NPs act as an antimicrobial agent, which increases the antimicrobial activity of CS/AV gels. Indeed, ZnO NPs have shown bactericidal and bacteriostatic activity by the generation of reactive oxygen species [32].

### 3.4. *In Vivo* Wound Healing Study

**Figure 3:**
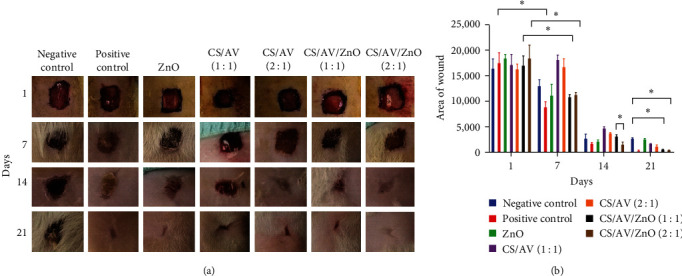
(a) Wound healing macroscopic images for 21 days in different groups. (b) Area of wound in treated animals at 1, 7, 14, and 21 days from wound creation. Data are represented as mean ± SD.  ^*∗*^ Denotes significant differences (*p*  < 0.05).

The wound healing efficiency of the prepared gels was investigated macroscopically over a period of 21 days. According to the results obtained from Figure 3, it was observed that after 7 days, the wound area was significantly reduced in the positive group. Additionally, the CS/AV/ZnO groups with ratios 1 : 1 and 2 : 1 also showed a significant reduction in wound area compared to the other treatment groups.

Recently, there has been a growing potential in utilizing nanotechnology for wound healing applications. In this context, various approaches have been studied for improving and accelerating of wound healing process [36, 37]. The utilization of ZnO NPs for wound healing has sparked interest as an effective approach to wound treatment. It could be attributed to their ability to modify immune responses and exhibited antimicrobial properties [31]. In this context, other studies have demonstrated that the incorporation of effective substances into the wound healing systems results in more appropriate antibacterial activity compared to free substances [38–40]. In the current study, after 21 days, the wound healing results demonstrated that CS/AV/ZnO gels were able to significantly decrease the wound area in contrast to the negative control and other treatment groups (*p*  < 0.05). The obtained results showed there is a significant difference between CS/AV/ZnO groups with 1 : 1 and 2 : 1 ratio and ZnO group after 21 days of treatment (*p*  < 0.05). The formulation of CS/AV/ZnO gels exhibited a similar performance to the positive control. Higher treatment rates in CS/AV/ZnO NPs compared to CS/AV and ZnO alone could be related to the incorporated NPs into CS/AV gel, which possibly results in higher healing activity based on their antimicrobial properties.

### 3.5. Histological Study

**Figure 4:**
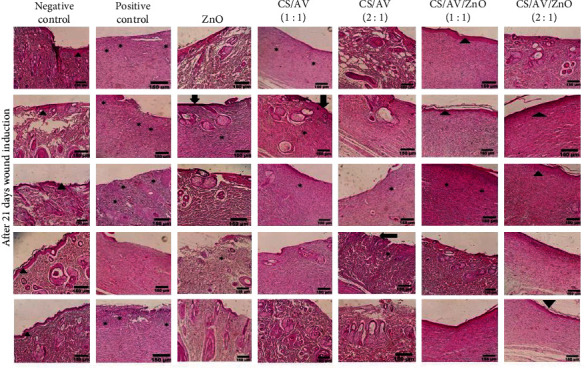
Histological assessment, 21 days after wound induction by H&E staining in different treatment groups, arrow heads indicate the re-epithelialization, and asterisks indicate the presence of inflammation.

**Figure 5:**
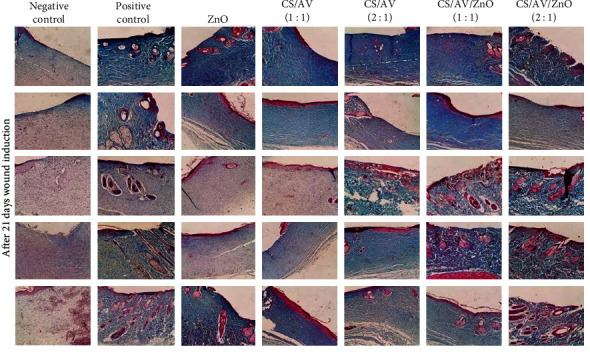
Histological assessment, 21 days after wound induction by Masson's trichrome (MT) staining in different treatment groups, blue staining displays the collagen fibers deposition.

Histological examination of the wounds was performed through H&E staining, as illustrated in Figure 4. In the group in which wounds were left untreated (negative control), the histopathological evaluation at 21 days posttreatment revealed the presence of inflammatory cell infiltration (polymorphonuclear cells) and the formation of granulation tissue. However, there was an absence in the formation of an epidermal layer and wounds were covered by a crusty scab. Conversely, the positive control exhibited completed epithelialization, and the inflammatory cell count was significantly reduced in contrast to the untreated control at the same time (Figure 4). In the ZnO treatment group, inflammation was observed in the wound area, but the inflammatory response was notably diminished, and epidermis formation was initiated. Furthermore, the CS/AV/ZnO groups with 1 : 1 and 2 : 1 ratios displayed evident epidermal proliferation and an increased epidermal layer 21 days posttreatment. The responses related to the inflammatory process and granulation tissue gradually diminished by employing these formulations (Figure 5).

Notably, the histopathological evaluation of the CS/AV/ZnO group with 2 : 1 ratio showed a significant reduction in inflammation. This particular group exhibited a closer resemblance to normal skin, with a thin epidermis and the presence of normal ridges and skin layers of regular thickness.

The scores for epitheliogenesis and collagen density at day 21 are displayed in Table 2. Movaffagh et al. [6] demonstrated that topical application of CS/AV hydrogel crosslinked with genipin had beneficial impacts in epithelization as well as more accelerated wound closing compared to CS and AV alone. Similarly, based on the current results, the highest score of epithelialization was observed in the CS/AV/ZnO groups with 1 : 1 and 2 : 1 ratios, which were comparable to the positive control group. Additionally, the results indicated that collagen deposition in the CS/AV/ZnO groups with 1 : 1 and 2 : 1 ratio was significantly higher compared to the other treatment groups after 21 days of treatment (Figure 4). Zhou et al. [41] demonstrated that the use of zinc crosslinked hydrogel can enhance the wound healing process by influencing collagen deposition, promoting the formation of granulation tissue, and stimulating the migration of fibroblasts. Furthermore, the hydrogel exhibited antibacterial activity, contributing to the overall effectiveness of the wound healing treatment. Their findings highlighted the potential of zinc crosslinked hydrogel as a promising approach to improve wound healing outcomes and offer potential benefits in the management of various wound types [41]. Similarly, in the current study employing ZnO NPs could improve the wound healing process by promoting both collagen deposition and also epithelialization.

In another study, the antibacterial activity of the alginate-CS matrix, along with AV and silver NPs has been evaluated. Their findings revealed that a combination of alginate, CS, AV gel, and silver NPs could be a potential alternative for antibacterial applications, particularly in the context of wound healing [42]. Furthermore, a study in 2018 demonstrated that AV gel in combination with CS NPs can induce a complete full-thickness wound healing activity at the end of 21 days. They concluded that AV with CS NPs thin film has positive effects on different wound healing stages, including reepithelization, granulation, and inflammation [43]. Their results showed that the combination of AV with CS NPs has reduced wound area significantly compared to AV or CS alone, which is consistent with the obtained results in this study. According to the findings at the end of 21 days, CS/AV/ZnO gels were able to significantly decrease the wound area when compared to the negative control (*p*  < 0.05). Furthermore, the CS/AV/ZnO gels exhibited a similar performance to the positive control. Moreover, the CS/AV/ZnO treatment groups with 1 : 1 and 2 : 1 ratios exhibited the highest collagen production and epithelization process compared to the other treatment group. In another study, the wound healing efficiency of a CS-poly(*N*-vinylpyrrolidone) (PVP) matrix incorporated with titanium dioxide (TiO_2_) NPs was evaluated. The results from this study demonstrated the great antibacterial properties of this dressing as well as being biocompatible due to its nontoxicity for NIH3T3 and L929 fibroblast cells [10].

In Vasile et al.'s [44] research, they demonstrated that a wound dressing formulation comprising CS gel loaded with a substantial concentration of ZnO NPs and coated with gentamicin displayed strong antibacterial efficacy. This remarkable impact was attributed to the intrinsic antibacterial properties of each component, which, when combined, resulted in a synergistic effect [44]. These findings suggest that the nanocomposite gel of CS/AV/ZnO NPs could be a potential candidate for addressing wound infections and promoting successful wound healing outcomes.

## 4. Conclusion

In the current study, CS/AV/ZnO NPs gel was fabricated successfully, and the characterization of the ZnO NPs was confirmed by XRD and FESEM. The CS/AV gel incorporated with ZnO NPs appears to be a promising and effective wound healing product. The obtained results showed there is a significant difference between CS/AV/ZnO groups with 1 : 1 and 2 : 1 ratio and other treatment groups after 21 days of treatment (*p*  < 0.05). The study demonstrated its positive effects on various aspects of the wound healing process, such as collagen deposition, epithelization, and inflammation. Moreover, this combination of ingredients may offer additional advantages, including the potential to prevent scar formation and microbial infections. Given the encouraging results from this study, we recommend further research and studies to explore the diverse applications of this gel in clinical settings. Conducting more comprehensive investigations will provide a deeper understanding of its therapeutic potential and expand its potential uses for different types of wounds and wound management approaches.

## Figures and Tables

**Table 1:** Characterization of different formulated gels.

Formulations	Appearance	Homogeneity	pH	Spreadability
CS/AV gel (1 : 1)	Yellow	Yes	5.5	Acceptable
CS/AV gel (2 : 1)	Yellow	Yes	5.5	Acceptable
CS/AV/ZnO gel (1 : 1)	Yellow	Yes	5.5	Acceptable
CS/AV/ZnO gel (2 : 1)	Yellow	Yes	5.5	Acceptable

**Table 2:** Histomorphometric analysis of different experimental groups.

Experimental groups	Epitheliogenesis score (mean ± SD)	Collagen density (%) (mean ± SD)
Negative control	0.8 ± 0.8	46.2 ± 3.7
Positive control	3.4 ± 0.8	81 ± 3.8
ZnO	2.4 ± 0.5	62 ± 6.3
CS/AV gel (1 : 1)	1.7 ± 0.5	56 ± 9.4
CS/AV gel (2 : 1)	1.7 ± 0.9	59.2 ± 10.7
CS/AV/ZnO gel (1 : 1)	3 ± 0.7	74.4 ± 3.9
CS/AV/ZnO gel (2 : 1)	3 ± 0.9	76.6 ± 3.3

## Data Availability

The data that support the findings of this study are available from the corresponding author upon request.
